# High-temperature resistive gas sensors based on ZnO/SiC nanocomposites

**DOI:** 10.3762/bjnano.10.151

**Published:** 2019-07-26

**Authors:** Vadim B Platonov, Marina N Rumyantseva, Alexander S Frolov, Alexey D Yapryntsev, Alexander M Gaskov

**Affiliations:** 1 Chemistry Department, Moscow State University, 1–3 Leninskie gory, Moscow, 119991 Russia; 2 Saint Petersburg State University, 7/9 Universitetskaya nab., Saint Petersburg, 199034 Russia; 3 Kurnakov Institute of General and Inorganic Chemistry of Russian Academy of Sciences, 31 Leninsky prospect, Moscow, 119991 Russia

**Keywords:** electrospinning, high temperature gas sensor, n–n heterojunction, ZnO/SiC nanocomposite

## Abstract

Increasing requirements for environmental protection have led to the need for the development of control systems for exhaust gases monitored directly at high temperatures in the range of 300–800 °C. The development of high-temperature gas sensors requires the creation of new materials that are stable under these conditions. The stability of nanostructured semiconductor oxides at high temperature can be enhanced by creating composites with highly dispersed silicon carbide (SiC). In this work, ZnO and SiC nanofibers were synthesized by electrospinning of polymer solutions followed by heat treatment, which is necessary for polymer removal and crystallization of semiconductor materials. ZnO/SiC nanocomposites (15–45 mol % SiC) were obtained by mixing the components in a single homogeneous paste with subsequent thermal annealing. The composition and microstructure of the materials were characterized by X-ray diffraction (XRD), scanning electron microscopy (SEM), Fourier-transform infrared spectroscopy (FTIR) and X-ray photoelectron spectroscopy (XPS). The electrophysical and gas sensing properties of the materials were investigated by in situ conductivity measurements in the presence of the reducing gases CO and NH_3_ (20 ppm), in dry conditions (relative humidity at 25 °C RH_25_ = 0) and in humid air (RH_25_ = 30%) in the temperature range 400–550 °C. The ZnO/SiC nanocomposites were characterized by a higher concentration of chemisorbed oxygen, higher activation energy of conductivity, and higher sensor response towards CO and NH_3_ as compared with ZnO nanofibers. The obtained experimental results were interpreted in terms of the formation of an n–n heterojunction at the ZnO/SiC interface.

## Introduction

The risk of air pollution is growing due to the development of new technologies in the chemical, metallurgical and food industries, the use of bio-fuels in the energy sector, modern waste treatment, and new automotive and aircraft engines [1–2]. Increasing requirements for environmental protection lead to the need for the development of control systems for exhaust gases that can directly monitor at high temperatures in the range of 300–800 °C. The composition of the main components of exhaust gas includes CO_2_, CO, SO_2_, H_2_S, NO_x_, C*_n_*H_2_*_n_*_+2_, and NH_3_. The ratio of these components depends primarily on the technology features and fuel type. High-temperature gas sensors are needed for local monitoring of pollution emissions, as well as for monitoring the complete combustion of fuel and controlling medium-temperature chemical and metallurgical processes [3–5]. The development of high-temperature gas sensors requires the creation of new materials that are stable at 300–600 °C, high humidity, and lack of oxygen. Nanostructured semiconductor oxides, such as SnO_2_, ZnO, WO_3_, and In_2_O_3_, that have been widely used in resistive gas sensors cannot be applied directly, primarily due to the drift of the sensor parameters at temperatures above 500 °C. The stability of nanostructured semiconductor oxides at high temperature can be enhanced by creating composite nanomaterials using highly dispersed silicon carbide (SiC). The unique physical and chemical properties of silicon carbide – wide band gap (*E*_g_ = 2.4–3.2 eV), high Debye temperature 1400 K, high thermal conductivity of 4.9 W/cm·K, low reactivity to oxygen and water vapor – ensure the stability of composite materials with respect to temperature, radiation, chemical and mechanical effects [6–7]. It has been shown that in MO/SiC nanocomposites containing metal oxide (MO) and nanostructured SiC, the presence of silicon carbide inhibits the growth of MO crystallites at high temperatures [8]. The difference in the adsorption properties, reactivity and electrical behavior of semiconductor oxides and silicon carbide, as well as possible chemical interactions on their interface, cause changes in the sensor performance of composite materials. The SiC-based materials in the form of planar Pt/MO/SiC heterostructures were intensively studied as sensitive elements of field-effect or Schottky diode gas sensors. These materials have a high sensitivity to hydrogen and hydrocarbons in the temperature range of 200–600 °C [9–14]. The resistive-type sensors based on MO/SiC composite materials have not been practically studied. A few works of the MO/SiC composite material based on highly dispersed silicon carbide [8,15] showed the stability of the material structure at 600 °C and its high response to carbon monoxide.

Electrospinning is inexpensive tool widely used today for preparation porous, ultrathin fibers of SiC and metal oxides as well as MO/SiC composites from polymer solutions [16–19]. The combination of unlimited length, highly porous microstructure, and high surface area come together to create ideal gas sensor materials.

In this work, we prepared ZnO/SiC nanocomposite materials by mixing and heat treatment of electrospun ZnO nanofibers and nanocrystalline silicon carbide of 3C-SiC polytype. The effect of silicon carbide on the structure and electrical properties of composite materials was studied using different techniques. The work is aimed at creating the resistive-type gas sensors based on ZnO/SiC composites and studying the sensor performance towards the main components of the exhaust gases CO and NH_3_ in air at a temperature range of 400–550 °C.

## Results and Discussion

**Figure 1:**
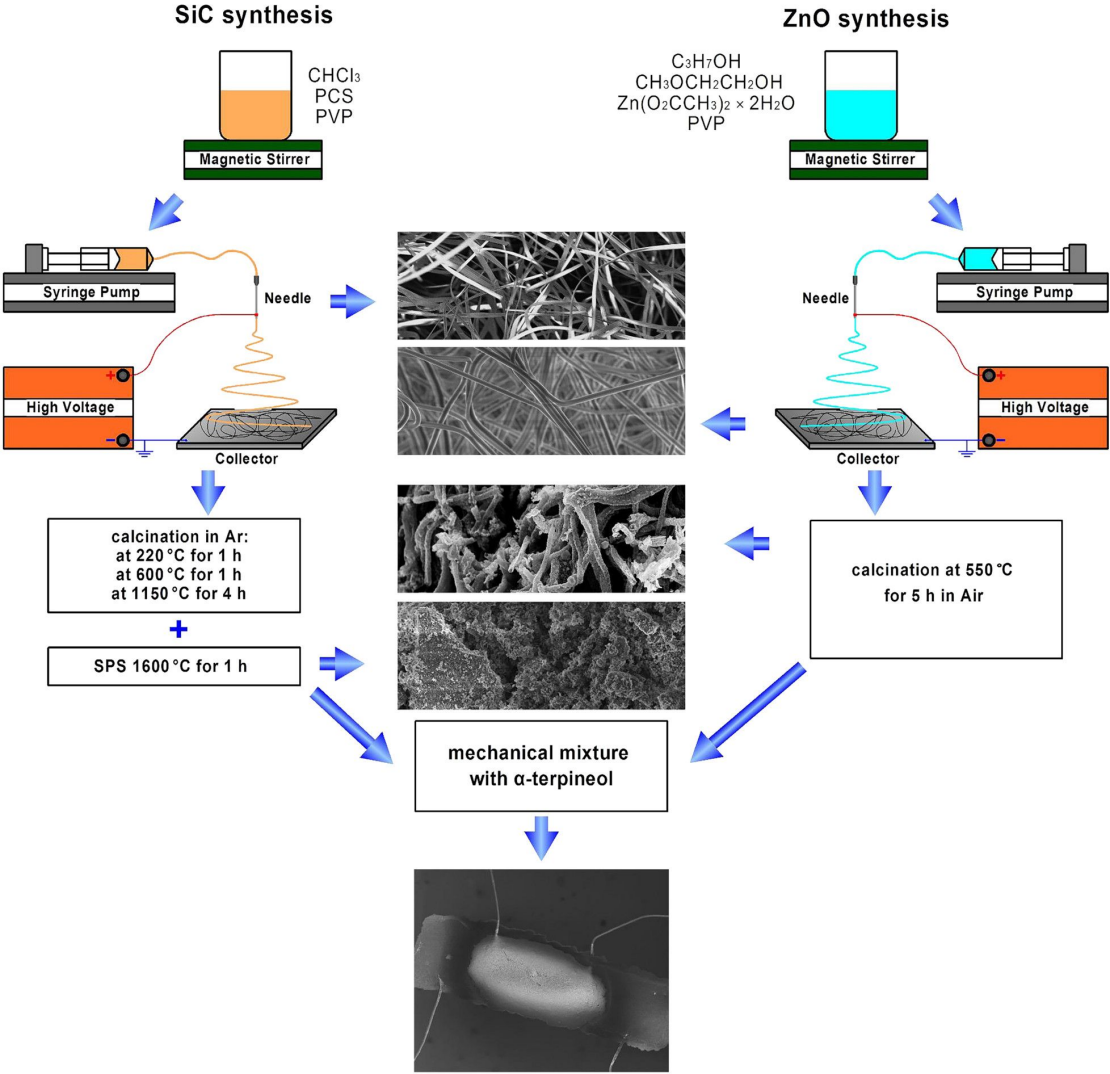
Synthesis scheme of nanocrystalline ZnO, SiC and ZnO/SiC nanocomposite materials.

The nanocomposite synthesis scheme is shown in Figure 1 and described in detail in the Experimental part. The characteristics of the synthesized materials are summarized in Table 1.

**Table 1:** Microstructure characteristics and electrophysical properties of ZnO nanofibers, ZnO/SiC nanocomposites and nanocrystalline SiC powder.

Sample	*С*_SiC_^a^, mol %	Phase composition, XRD	*d*_XRD_^b^, nm	*S*_BET_^c^, m^2^/g	*R*_air_^d^, Ohm (400 °C)	*E*_a_^e^, eV

ZnO	0	ZnO	18 ± 2	10 ± 1	8.6 × 10^5^	0.40 ± 0.04
ZnO/SiC_15	15	ZnO/SiC	18 ± 2/25 ± 3	–	4.6 × 10^6^	0.71 ± 0.06
ZnO/SiC_30	30	ZnO/SiC	18 ± 2/25 ± 3	–	7.0 × 10^6^	0.73 ± 0.09
ZnO/SiC_45	45	ZnO/SiC	18 ± 2/25 ± 3	–	1.5 × 10^7^	0.78 ± 0.07
SiC	100	SiC	27 ± 3	6 ± 1	8.5 × 10^9^	–

^a^SiC content; ^b^crystallite size estimated from the broadening of the (100) ZnO and (111) 3C-SiC reflections using the Scherrer formula; ^c^specific surface area; ^d^resistance in dry air at 400 °C; ^e^activation energy of conductivity in the temperature range 400–500 °C.

**Figure 2:**
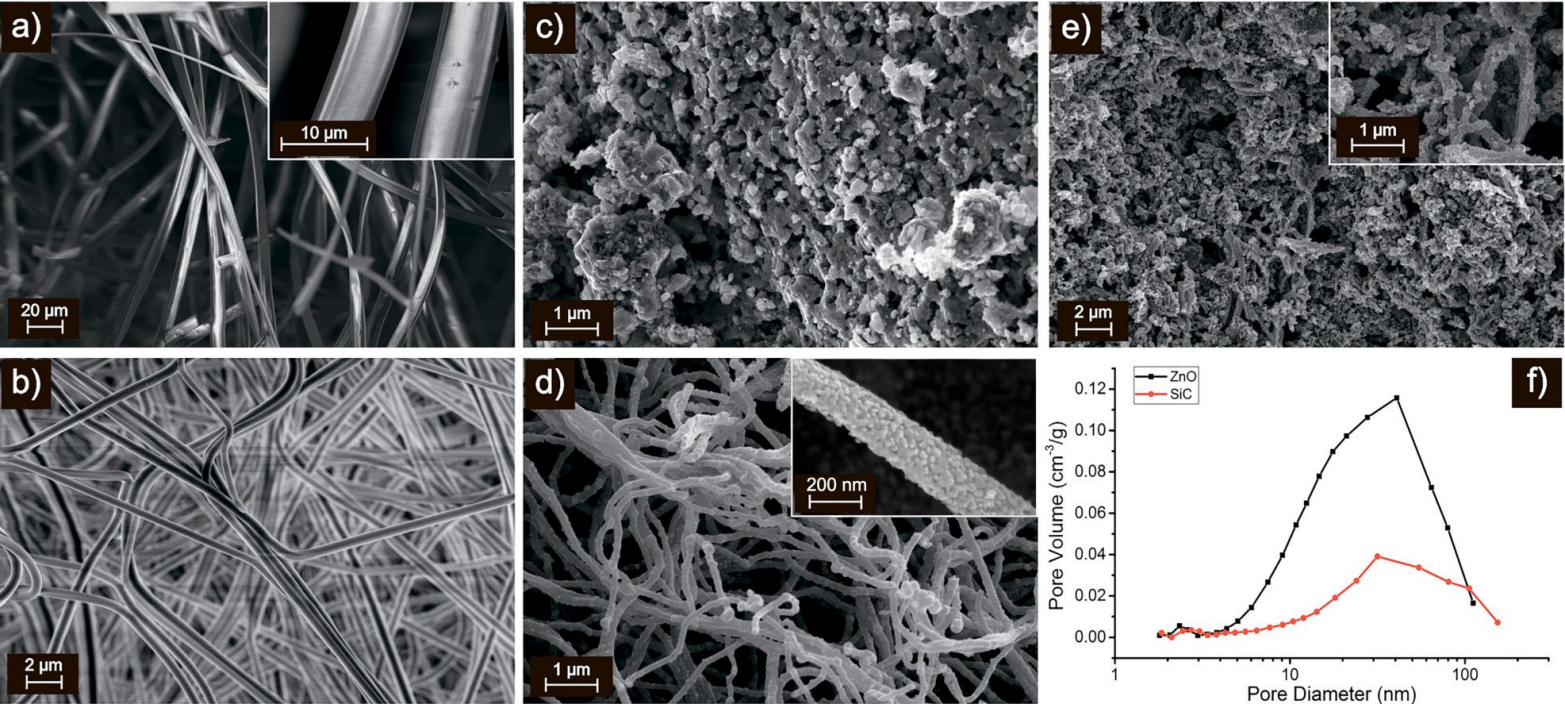
SEM micrographs of polymer nanofibers containing polycarbosilane (a) and zinc acetate (b). SEM micrographs of annealed SiC (c) and ZnO (d). (e) SEM micrograph of ZnO/SiC_45 nanocomposite. (f) Pore size distribution of annealed ZnO and SiC.

Figure 2 shows the scanning electron microscopy (SEM) micrographs of SiC (Figure 2a,c) and ZnO (Figure 2b,d) nanofibers in a polymer matrix (Figure 2a,b) and after annealing (Figure 2c,d). The polymeric fibers containing polycarbosilane (Figure 2a) are tapered with a width of 8–10 μm and a thickness of about 200 nm. Exposure to high temperature and pressure, which is necessary for the formation of crystalline SiC, leads to the destruction of fibers and the formation of porous powders (Figure 2c) with an average pore diameter of 30 nm (Figure 2f). The polymeric fibers containing zinc acetate (Figure 2b) are cylindrical wires about 500 nm in diameter. The ZnO nanofibers obtained after annealing (Figure 2d) consist of polycrystalline wires with an average diameter of 150 nm formed by nanocrystals about 20–30 nm in size. The average pore diameter, estimated from the data of low-temperature nitrogen adsorption, was 50 nm (Figure 2f). In the ZnO/SiC nanocomposites (Figure 2e), formed from ZnO nanofibers and SiC powder by mixing components in a single homogeneous paste with subsequent annealing at 550 °C, the quasi-one-dimensional structure of ZnO wires is retained.

**Figure 3:**
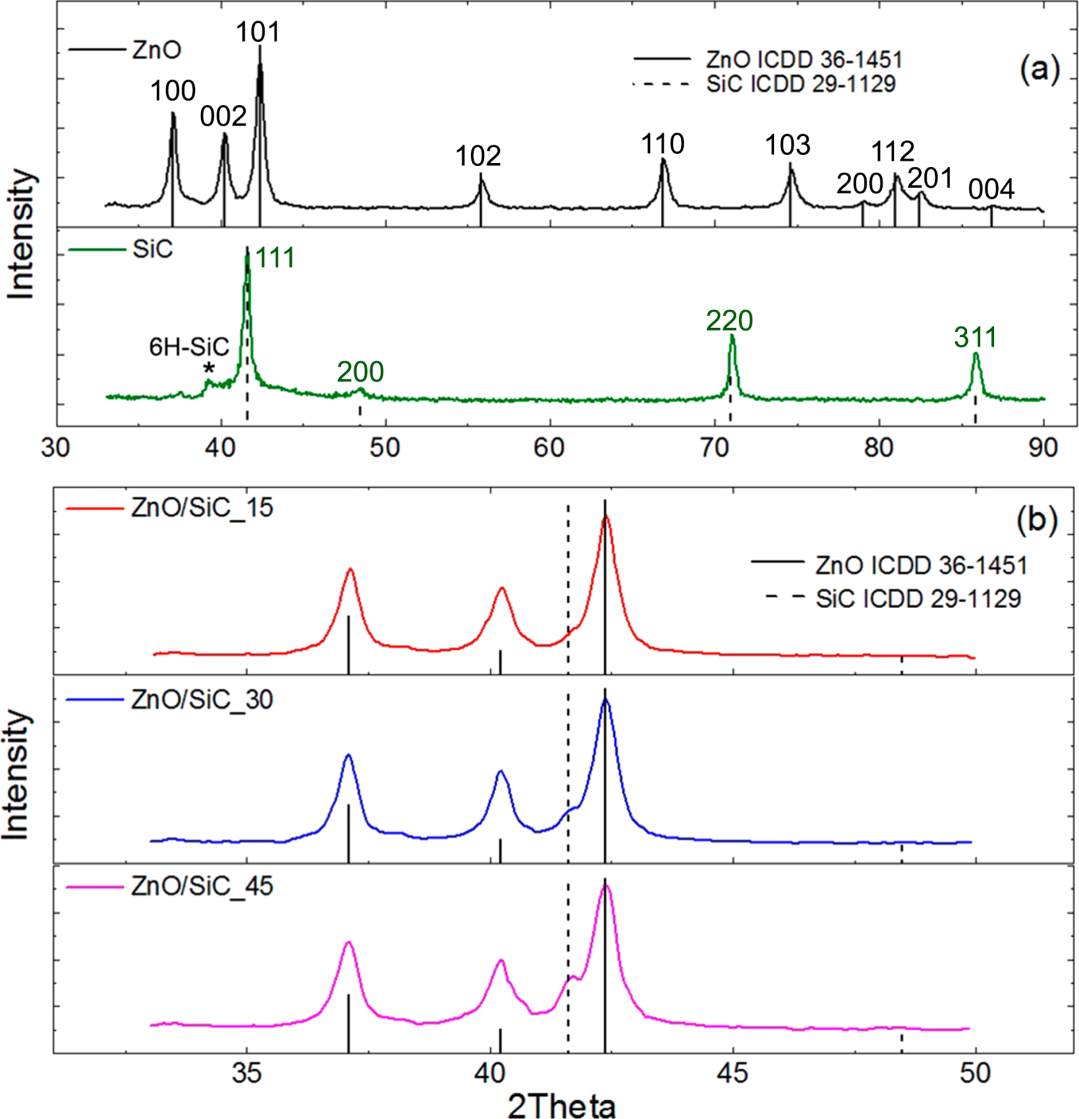
X-ray diffraction patterns of (a) ZnO nanofibers and nanocrystalline SiC and (b) ZnO/SiC nanocomposites. The vertical solid and dotted lines correspond to ICDD 36-1451 (ZnO, wurtzite) and ICDD 29-1129 (SiC-3C polytype) references, respectively.

The X-ray diffraction data indicate (Figure 3a) that the annealing of polymer fibers leads to the formation of crystalline phases of ZnO (wurtzite, ICDD 36-1451) and SiC (3C polytype, ICDD 29-1129). The crystallite size (*d*_XRD_), estimated from the broadening of (100) ZnO and (111) 3C-SiC diffraction peaks, as determined by the Scherrer formula, is consistent with the size of crystalline particles from SEM analysis. The diffraction patterns of ZnO/SiC nanocomposites contain diffraction maxima of both crystalline phases (Figure 3b), and the intensity of the SiC peaks naturally increases with increasing silicon carbide content in the nanocomposites.

**Figure 4:**
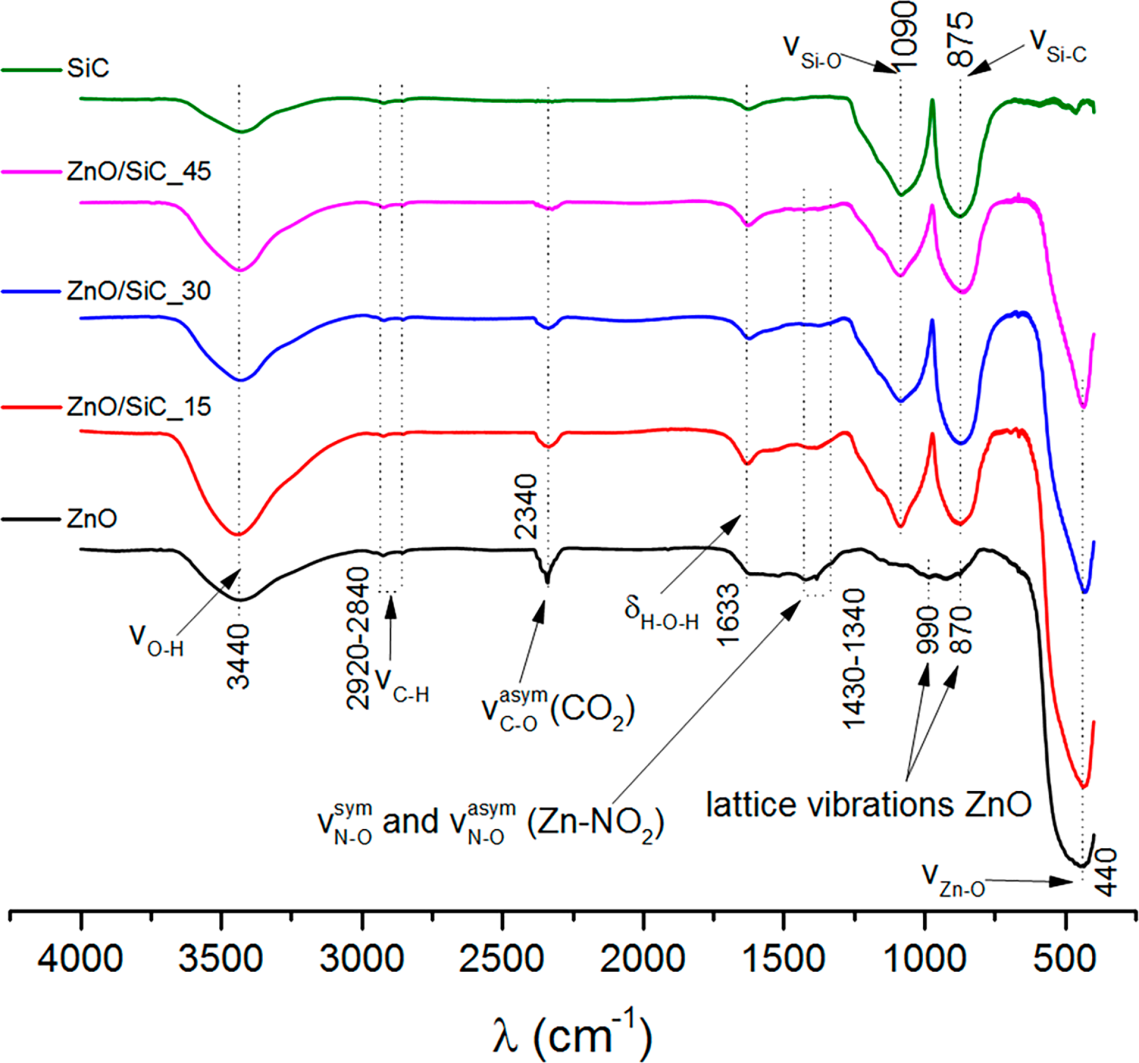
FTIR spectra of ZnO nanofibers, nanocrystalline SiC and ZnO/SiC nanocomposites.

The study of the surface composition of the synthesized materials was carried out using FTIR and XPS methods. Figure 4 shows the IR absorption spectra of ZnO, SiC, and ZnO/SiC nanocomposites. The spectrum of zinc oxide contains an intense broad signal, corresponding to the stretching vibrations of Zn–O bonds (635–400 cm^−1^). The above spectrum also shows the signals from the multi-phonon vibrational modes of the ZnO lattice (990 and 870 cm^−1^). In accordance with the literature data, such oscillations do not appear at 78 K, but are noticeable at room temperature [20]. Nitro and nitrite groups [21–22] formed during the decomposition of PVP are also present on the surface of zinc oxide, as evidenced by the appearance of IR signals in the 1430–1260 cm^−1^ region, corresponding to the symmetric and asymmetric oscillations of the N–O bond. A broad peak in the region of 3750–3000 cm^−1^ is due to the stretching vibrations of hydroxy groups on the ZnO surface. The deformation vibrations of adsorbed water molecules are recorded at 1640 cm^−1^. In addition to these, the spectrum contains peaks related to the vibrations of the C–O bond in CO_2_ molecules adsorbed on the ZnO surface (2430–2320 cm^−1^) and C–H bonds (2920–2840 cm^−1^) in the residues of the organic components used in the synthesis of ZnO nanofibers.

The FTIR absorption spectrum of the SiC sample contains two intense peaks with absorption maxima at 900 cm^−1^ and 1067 cm^−1^, corresponding to the stretching vibrations of the Si–C and Si–O bonds, respectively [23]. This indicates the formation of an amorphous SiO_2_ shell on the surface of SiC nanoparticles, which does not appear on the diffraction patterns of the samples. In addition to these absorption lines, the spectrum contains the signals corresponding to O–H vibrations of surface hydroxy groups, deformation vibrations of adsorbed water molecules, and C–H bonds in the residues of organic components. All these oscillations are also present in the FTIR spectra of ZnO/SiC nanocomposites with the intensity ratio corresponding to the molar ratio of ZnO and SiC. Any additional vibrational modes do not arise in the FTIR spectra of ZnO/SiC nanocomposites.

**Figure 5:**
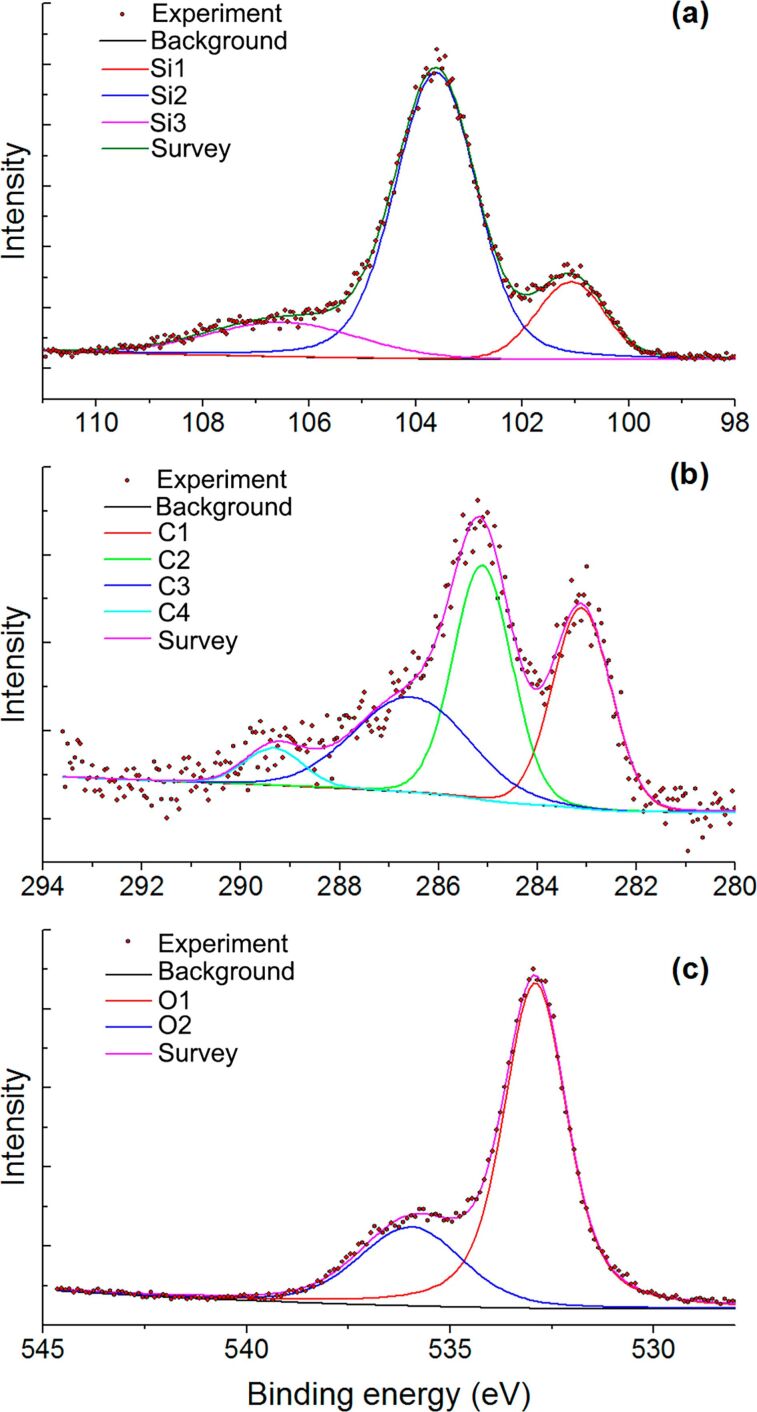
X-ray photoelectron spectra of SiC in the Si 2p (a), C 1s (b), and O 1s (c) regions.

**Figure 6:**
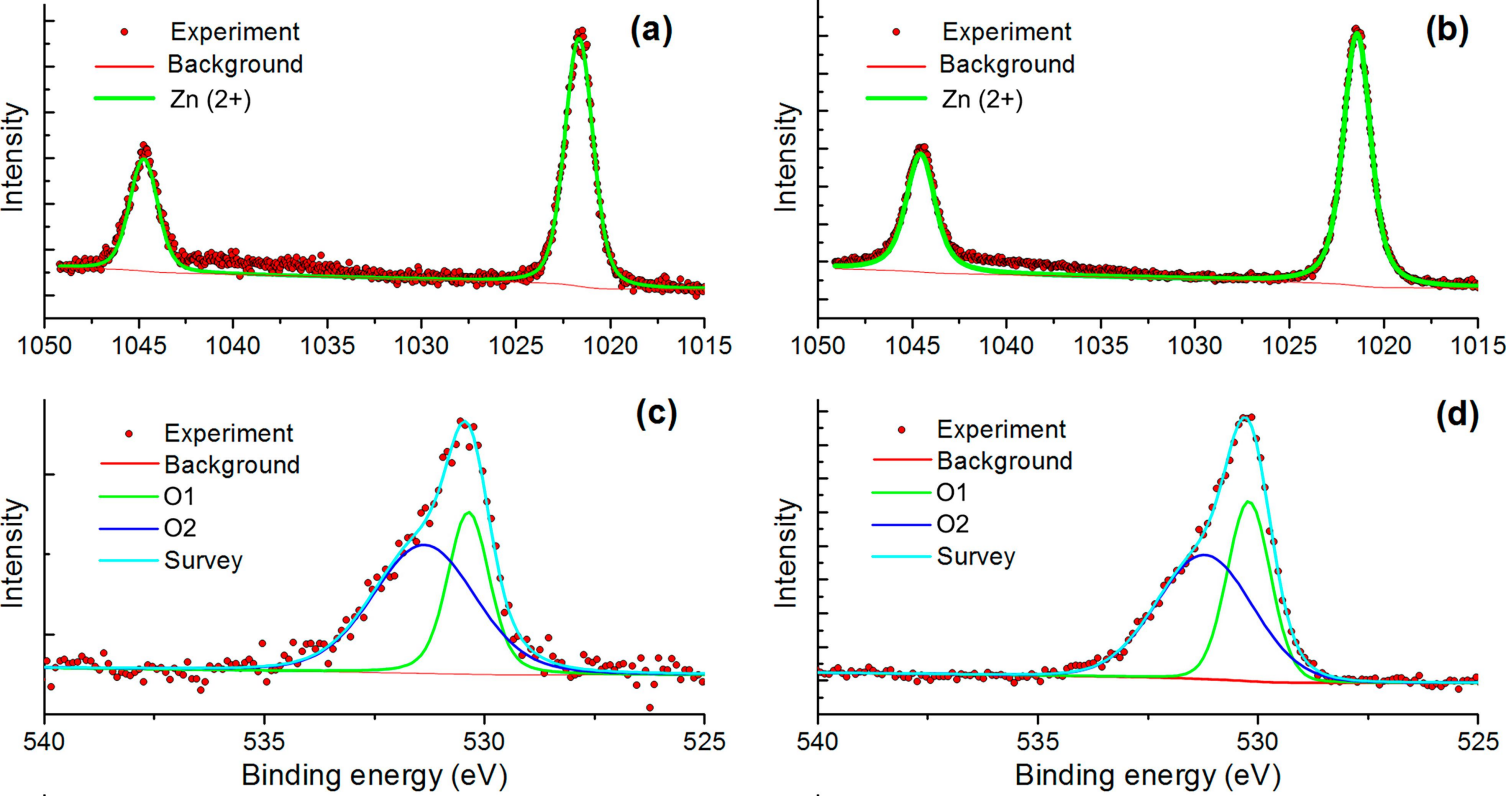
X-ray photoelectron spectra of the ZnO/SiC_15 nanocomposite (a, c) and ZnO nanofibers (b, d) in the Zn 2p (a, b) and O 1s (c, d) regions.

To reveal the possible interactions between SiC and ZnO nanoparticles, and to shed light on the surface composition of the materials, we used X-ray photoelectron spectroscopy (XPS). Figure 5 and Figure 6 show the XPS spectra of ZnO, SiC and the ZnO/SiC_15 nanocomposite in the Zn 2p, O 1s, Si 2p, and C 1s binding energy regions. The survey spectra are provided in Supporting Information File 1 (Figure S1). For the SiC sample, it was found that the Si 2p region contains three components at 100.6 (Si1), 103.0 (Si2), and 106.3 (Si3) eV (Figure 5a). The first one corresponds to silicon carbide, while the second one refers to silicon oxide [24]. A weak third component may be associated with Si–O_2_ bonds [19]. The formation of silicon oxide is also observed in the photoelectron spectrum in the O 1s region, containing two components at 532.9 (O1) and 536 (O2) eV. The first component is assigned to the oxygen bonded to two silicon atoms [24] while the second one may be associated with the chemisorbed oxygen (Figure 5c). The presence of an Si–O component in the O 1s spectrum is consistent with the results from IR spectroscopy. The carbon in silicon carbide is also found to be oxidized. The spectrum of the C 1s region contains four components at 283.1 (C1), 285.1 (C2), 286.5 (C3), 289.3 and (C4) eV, which correspond to carbide in SiC, amorphous carbon, C–O and ether groups, respectively [24] (Figure 5b). For ZnO nanofibers and the ZnO/SiC_15 nanocomposite, the XPS spectra in the Zn 2p region depicted in Figure 6a,b contain only one component related to Zn in (+2) oxidation state. The XPS spectra in the O 1s region (Figure 6c,d) contain two components. The first one (O1) at 530.2 eV corresponds to the lattice oxygen in the ZnO phase, while the high-energy component (O2) at 531.2 eV is assigned to the different oxygen-containing species on the zinc oxide surface, which include hydroxy groups and different forms of chemisorbed oxygen [25–28]. No changes were detected in the Zn 2p XPS spectrum of ZnO/SiC nanocomposites compared with the similar spectrum of ZnO. At the same time, the ratio O1/O2 = 0.75 in the O 1s spectrum of the ZnO/SiC_15 composite is reduced as compared with that in the ZnO O 1s spectrum (O1/O2 = 0.85), which may be due to the increase in the concentration of oxygen surface species. The contribution from the Si–O component (532.9 and 536 eV, Figure 5c) in the O 1s spectrum of ZnO/SiC_15 is negligible. The XPS spectrum in Si 2p region, depicted in Figure S2 (Supporting Information File 1), proves the presence of silicon in the ZnO/SiC_15 nanocomposite.

**Figure 7:**
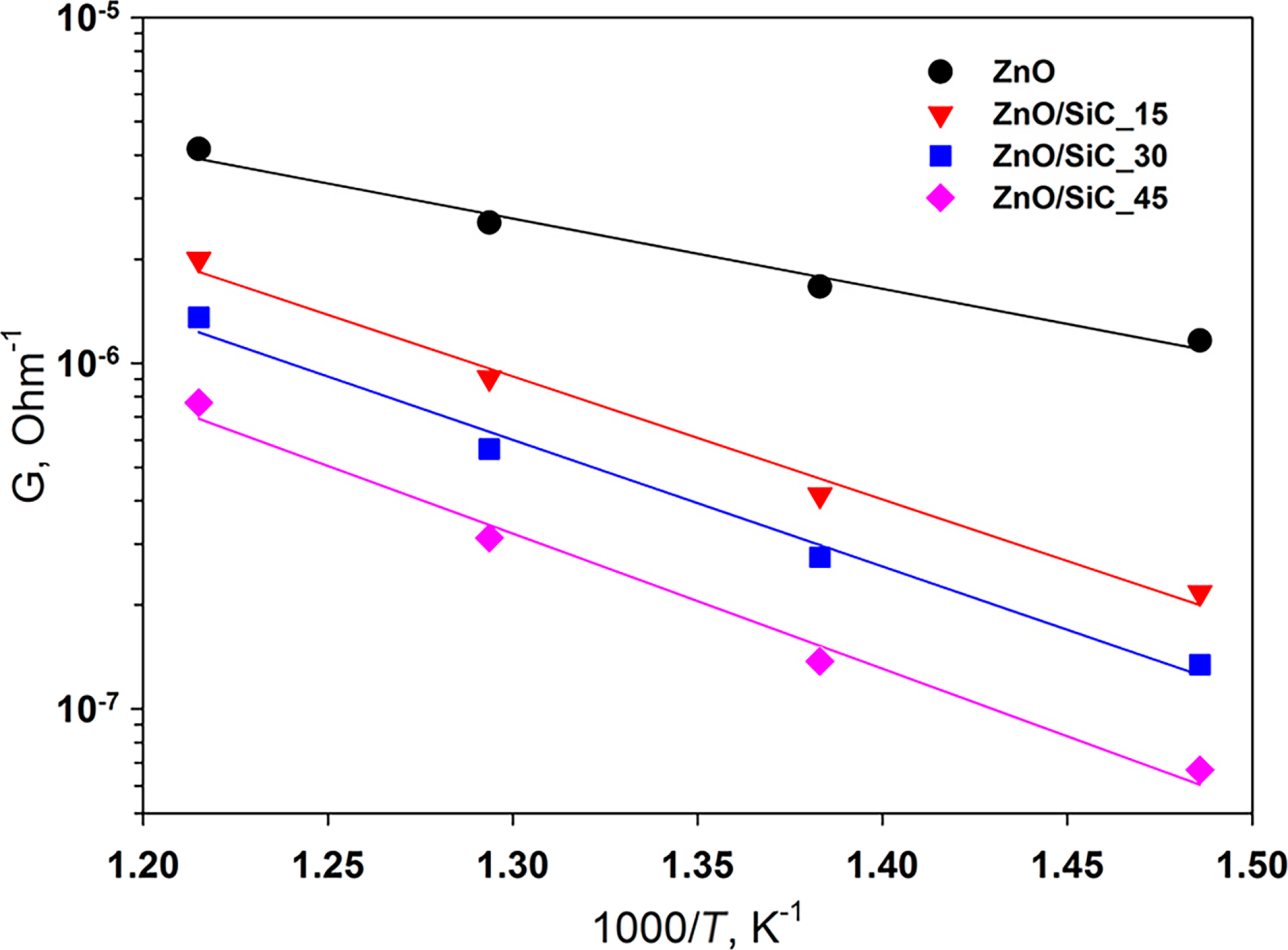
The conductance, *G*, of ZnO nanofibers and ZnO/SiC nanocomposites in the temperature range 400–550 °C.

The formation of nanocomposites is accompanied by a significant increase in the electrical resistance of the material, in comparison with ZnO nanofibers, over the entire temperature range studied (Table 1). The resistance of SiC under these conditions is in the range of 10^9^–10^11^ Ohm, which corresponds to the upper limit of the measurement range of the used setup. In the temperature range *T* = 400–550 °C, the conductivity of ZnO nanofibers and ZnO/SiC nanocomposites has an activation character (Figure 7). From the Arrhenius equation ln*G* = *E*_a_/*k*_B_*T*, where *G* is the material conductance, *k*_B_ is the Boltzmann constant, the values of the activation energy *E*_a_ were calculated. For ZnO nanofibers, *E*_a_ = 0.40 ± 0.04 eV. This value lies within the error with the potential barrier at the grain boundaries *eV*_s_ (the surface potential barrier energy between particles of nanocrystalline zinc oxide) determined by the method of temperature-stimulated conductance measurements [29–30] as *eV*_s_ = 0.44 eV at *T* = 500 °C [31]. The creation of ZnO/SiC nanocomposites leads to an increase in the activation energy of conductivity up to 0.71–0.78 eV, and the value of *Е*_a_ does not depend on the SiC content in nanocomposites (within the error, Table 1). The growth of the electrical resistance and *E*_a_ can be associated with an increase in the concentration of surface oxygen species (confirmed by XPS), which form different acceptor levels at the ZnO surface and at the ZnO/SiC heterojunction.

**Figure 8:**
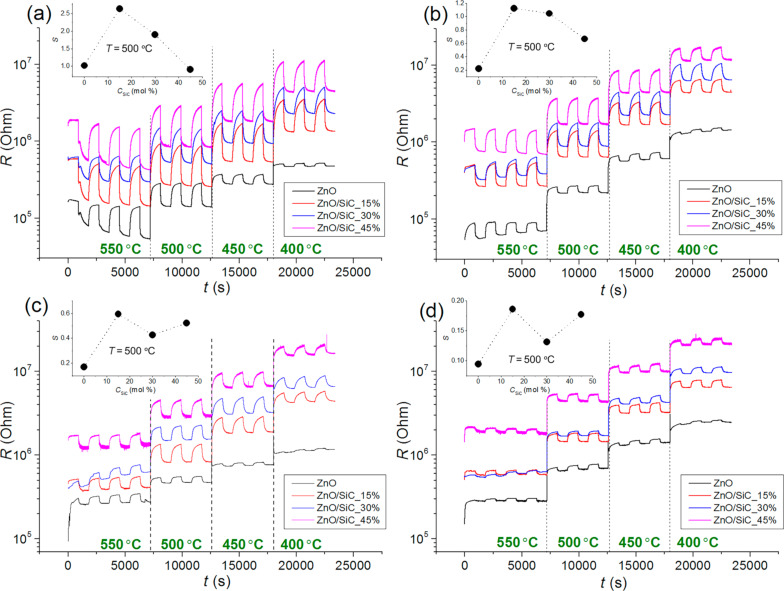
Change in the resistance of ZnO nanofibers and ZnO/SiC nanocomposites with a periodic change in the composition of the gas phase in the presence of 20 ppm NH_3_ (a,b) and 20 ppm CO (c,d) in dry air (a,c) and at relative humidity RH_25_ = 30% (b,d). Insets: Sensor response dependence on SiC content in nanocomposites.

The sensor properties of the synthesized materials were investigated by in situ conductivity measurements. Figure 8 shows the change in the resistance of ZnO nanofibers and ZnO/SiC nanocomposites with a periodic change in the composition of the gas phase in the presence of NH_3_ (Figure 8a,b) and CO (Figure 8c,d) in dry air (Figure 8a,c) and at relative humidity RH_25_ = 30% (at 25 °C, Figure 8b,d). In all cases, in the presence of a reducing gas, CO or NH_3_, a decrease in the material resistance is observed due to reaction of the target gases with the oxygen chemisorbed on the surface of n-type semiconductor materials:

[1] $$\beta \cdot {\text{CO}}_{\text{(gas)}}+{\text{O}}_{\beta \text{(ads)}}^{-\alpha }\to \beta \cdot {\text{CO}}_{\text{2(gas)}}+\alpha \cdot e^{-}$$

[2] $$2\beta \cdot {\text{NH}}_{\text{3(gas)}}+3{\text{O}}_{\beta \text{(ads)}}^{-\alpha }\to \beta \cdot {\text{N}}_{\text{2(gas)}}+3\beta \cdot {\text{H}}_{2}{\text{O}}_{\text{(gas)}}$$

where CO_(gas)_, NH_3(gas)_ are molecules of carbon monoxide and ammonia in the gas phase, 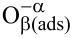 is a particle of chemisorbed oxygen, *e*^−^ is an electron released into the conduction band; CO_2(gas)_, N_2(gas),_ H_2_O_(gas)_ are the molecules of the reaction products desorbed from the surface of the material to the gas phase.

The data obtained allowed us to calculate the value of the sensor response as

**Figure 9:**
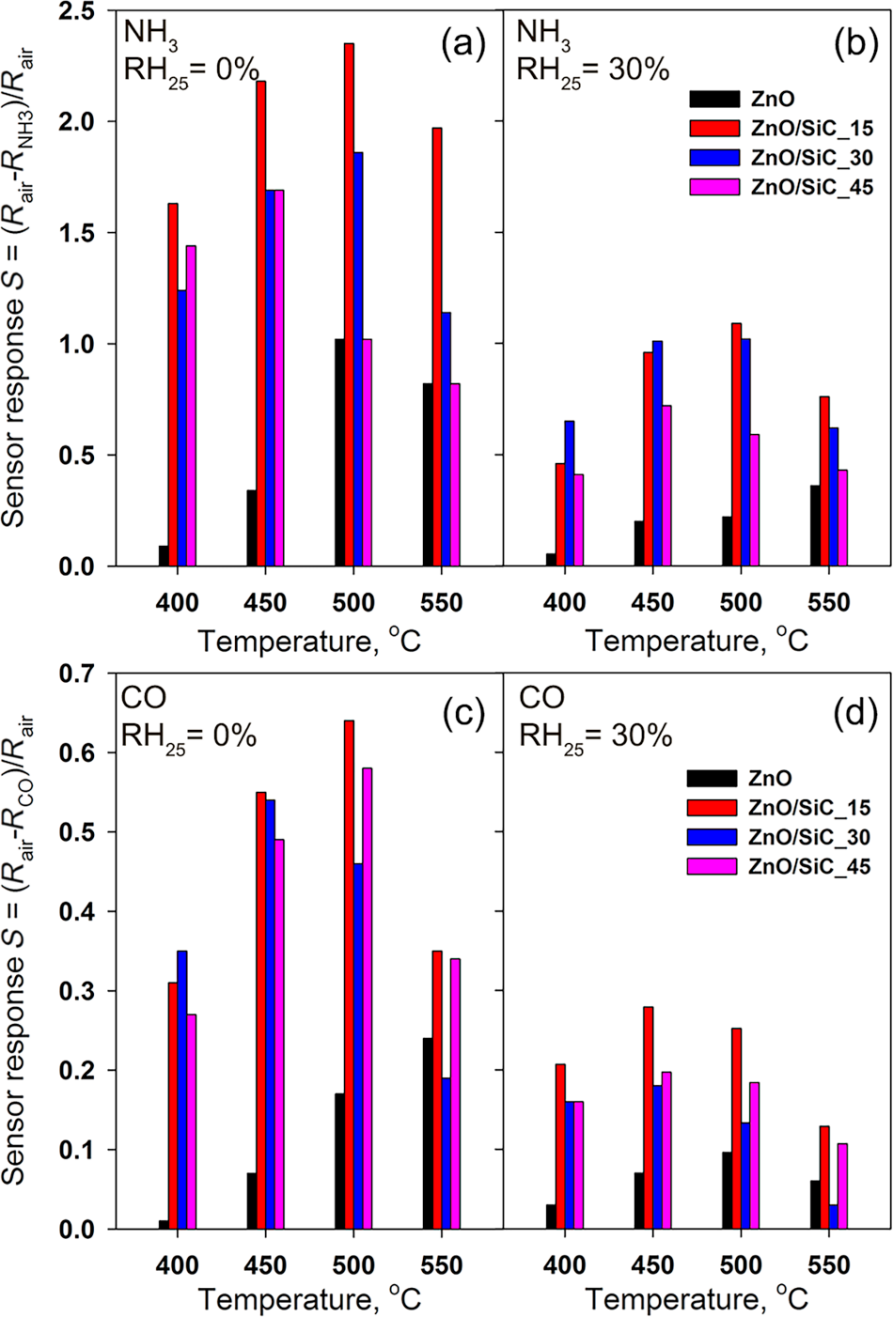
Temperature dependence of the sensor response of ZnO nanofibers and ZnO/SiC nanocomposites towards 20 ppm NH_3_ (a,b) and 20 ppm CO (c,d) in dry air RH_25_ = 0% (a,c) and at relative humidity RH_25_ = 30% (b,d).

[3] $$S=\frac{R_{\text{air}}-R_{\text{gas}}}{R_{\text{air}}},$$

where *R*_air_ is the resistance of the material in background air, and *R*_gas_ is the resistance of the material in the presence of the target gas (СО or NH_3_). The temperature dependence of the sensor response is shown in Figure 9. The maximum sensor response of ZnO nanofibers is observed at operating temperatures in the range of 500–550 °C, and in the case of ZnO/SiC nanocomposites, at *T* = 450–500 °C for both reducing gases. In both cases, the formation of ZnO/SiC nanocomposites leads to an increase in the sensor response compared to the bare ZnO nanofibers. The nanocomposites ZnO/SiC_15 and ZnO/SiC_30 demonstrate the highest values of the sensor response. A further increase in the SiC content leads to an increase in resistance and a decrease in the sensor response of the nanocomposites. Thus, from the point of view of the measured values of the sensor response and the base resistance in the temperature range of 400–500 °C, the composition of the material, which corresponds to 15 mol % SiC, is optimal. An increase in air humidity up to RH_25_ = 30% leads to an approximately two-fold decrease in the sensor response to CO and NH_3_.

The literature data characterizing conductometric gas sensors based on different MO/SiC systems are summarized in Table 2. It should be noted that there are few examples found in the literature [32–34], and all the found sources consider different gases. This does not allow for a correct comparison of the sensitivity of the materials obtained in this work with the analogues described in the literature.

**Table 2:** Sensor response of conductometric gas sensors based on different MO/SiC sensitive materials.

Sensor material	Gas	Concentration, ppm	Temperature, °C	Relative humidity, %	Sensor response 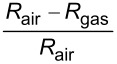	Ref.

ZnO/SiC	CO	20	500	30	1.1	this work
	NH_3_	20	450	30	0.27	
SnO_2_/SiC	H_2_	100	500	n/a	3.7	[32]
	xylene	100	500	n/a	0.8	
	acetone	100	500	n/a	1.8	
	isopropanol	100	500	n/a	1.6	
	methanol	100	500	n/a	2.1	
	ethanol	100	500	n/a	6.2	
SnO_2_/SiC	NH_3_	50	RT + UV^a^	30	0.2	[33]
	NO_2_	5	RT + UV	30	0.12	
WO_3_/SiC	H_2_	20000	350	n/a	0.9	[34]

^a^At room temperature under UV light activation.

**Figure 10:**
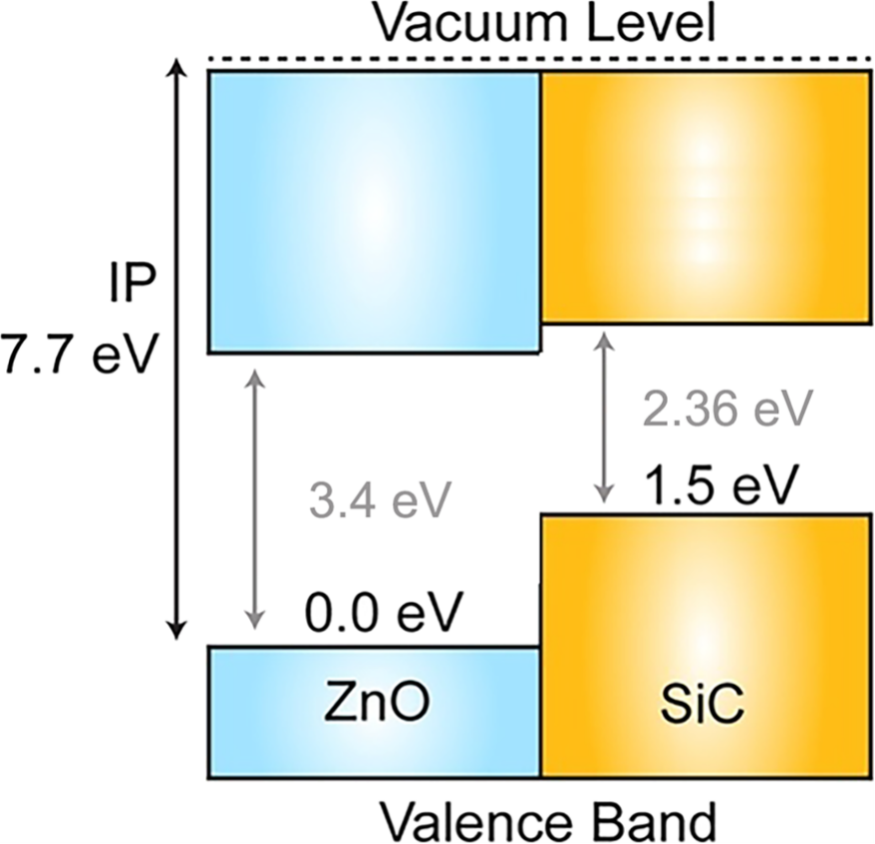
Estimated band alignment of the wurtzite ZnO and 3C-SiC phases. Adapted from [37] with permission from the American Chemical Society, copyright 2013.

The observed effect of SiC on the sensor response of ZnO nanofibers toward CO and NH_3_ should be considered within the framework of a model involving the formation of n–n heterocontacts at the ZnO/SiC interface [35]. According to a previous report [36] the conduction band minimum (CBM) of n-type ZnO lies 0.4 eV lower than the CBM of n-type SiC. The calculated band alignment of the wurtzite (2H) ZnO and SiC phases is presented in previous reports [37–38]. Taking into account the difference in the band gap (*E*_g_) of 2H-SiC (*E*_g_ = 3.3 eV [39]) and 3C-SiC (*E*_g_ = 2.36 eV [40]) polytypes, and assuming that the position of the valence band for these polytypes does not vary significantly, we constructed a diagram of the band alignment for ZnO and 3C-SiC phases (Figure 10). The estimated CBM position of wurtzite ZnO is 0.46 eV lower than that of 3C-SiC. The interface of an n–n junction transfers electrons into the lower energy conduction band [35]. The “accumulation layer” formed in this way can be depleted by subsequent oxygen adsorption on an enriched electron ZnO surface, increasing the potential energy barrier and enhancing the response formed due to reactions (Equation 1 and Equation 2). The decrease in the sensor response observed for all the samples with an increase in the concentration of water vapor in the gas phase may be due to the competition of oxygen and water molecules for the same adsorption centers on the ZnO surface [41].

## Conclusion

ZnO/SiC nanocomposites based on ZnO nanofibers (wurtzite) and nanocrystalline SiC (3C polytype), obtained by the electrospining method, were investigated as sensitive materials for high-temperature resistive gas sensors. The introduction of SiC increased the sensitivity of ZnO nanofibers towards the reducing gases CO and NH_3_ in the temperature range of 400–550 °C. This effect was accompanied by the increase in the activation energy of conductivity in this temperature range. The results obtained were interpreted in the context of the assumption of the formation of an n–n heterojunction at the ZnO/SiC interface, resulting in electron transfer from SiC to ZnO. The increase in the concentration of electrons in the near-surface layer of ZnO leads to an increase in the concentration of chemisorbed oxygen on its surface, which was confirmed by XPS. In turn, this determines an increase in the activation energy of conductivity and causes an increase in the sensor response of ZnO/SiC nanocomposites compared with ZnO nanofibers.

## Experimental

### Materials synthesis

Nanocrystalline silicon carbide, SiC, and zinc oxide, ZnO, were prepared separately by electrospinning of polymer solutions followed by heat treatment in order to remove the polymer and crystallize the semiconductor material. The annealing conditions for polymer decomposition were determined by thermal analysis.

### Fabrication of nanocrystalline SiC

Polycarbosilane (PCS) was used as a precursor. In a typical procedure, 1 g of PCS was dissolved in 10 mL of chloroform. After the PCS completely dissolved, 1 g of polyvinylpyrrolidone (PVP, *M* = 1 300 000) was added. The mixture was actively stirred for 5 h at 40 °C. The polymer solution was loaded in a plastic syringe with a metal needle (G21) with an internal diameter of 510 μm. The electrospinning was carried out at the conditions of 3 mL/h solution feed rate, with 150 mm distance and 6 kV voltage between the needle and metal collector. The formed fibrous tissue was collected in an alundum Al_2_O_3_ crucible and annealed stepwise in an argon atmosphere at 220 °C (2 h, heating rate 1 K/min), 600 °C (2 h, heating rate 1 K/min), and finally at 1150 °C (6 h, heating rate 2 K/min). The obtained amorphous SiC was additionally annealed using the spark plasma sintering (SPS) method on a Spark plasma sintering system (LABOX-625) at a temperature of 1600 °C for 1 h under vacuum. As a result, 3C-SiC nanofibers with a cubic structure were obtained. The final annealing step was performed in air at 700 °C for 1 h to remove the rest of the carbon.

### Fabrication of ZnO nanofibers

Zinc acetate (Zn(CH_3_COO)_2_·2H_2_O) was used as a precursor. In a typical procedure, 200 mg of zinc acetate was dissolved in 10 mL of mixed (1:1) solvent composed of 2-methoxyethanol and isopropanol. After complete dissolution of zinc acetate, 900 mg of PVP was added and the mixture was actively stirred for 5 h at 40 °C. The electrospinning of the polymer solution was carried out at the conditions of 1 mL/h solution feed rate, with 125 mm distance and 12 kV voltage between the needle and metal collector. The fibrous material was collected and heated at 550 °С (5 h, heating rate 1 K/min) in air in order to remove the polymer and crystallize the ZnO.

### Fabrication of gas sensors

ZnO/SiC nanocomposites containing 0, 15, 30, 45 and 100 mol % SiC were prepared by mixing components in a single homogeneous paste using a solution of α-terpineol in ethanol as a binder. The sensors were fabricated by thick film technology via drop-deposition of the paste onto alumina micro-hotplates provided with vapor-deposited Pt contacts (0.3 × 0.2 mm^2^) separated by a 0.2 mm gap and with embedded Pt-meanders. The paste was dried at room temperature in ambient air and then calcined at 250 °C in purified air for 20 h to remove the binder. The thick sensing layer was about 1 × 0.5 mm in size with the thickness of 5–7 μm. The list of the samples is given in Table 1.

### Materials characterization

The phase composition was determined by X-ray diffraction (XRD) using a DRON-3 diffractometer (radiation Co Kα, λ = 1.7903 Å). The crystallite size (*d*_XRD_) of SiC and ZnO phases in nanofibers was estimated from the broadening of the (100) ZnO and (111) 3C-SiC XRD peaks using the Scherrer formula. The measurements of the specific surface area (*S*_BET_) and analysis of the porosity of the samples were carried out by the method of low-temperature nitrogen adsorption on an ASAP 2010 instrument (Micromeritics). Prior to this, all samples were evacuated at a temperature of 300 °C to 4 × 10^−1^ Pa for 3 h. Based on the nitrogen adsorption isotherms obtained, the specific surface area, volume, and average pore size were calculated using BET (Brunauer–Emmett–Teller) and BJH (Barret–Johner–Halenda) models. The morphology of the nanofibers was studied by scanning electron microscopy (SEM) using a Carl Zeiss NVision 40 electron microscope with an intra-lens detector at an accelerating voltage of 5 kV. The IR spectra (FTIR) of the ZnO/SiC nanocomposites were taken on a Spectrum One (Perkin Elmer) spectrometer in transmission mode within the range 400–4000 cm^−1^ with 1 cm^−1^ steps. The XPS experiments were performed using an Axis Ultra DLD (Kratos) X-ray photoelectron spectrometer, equipped with a monochromatic Al Kα source. XPS spectra of core levels were fitted by Gaussian/Lorentzian convolution functions with simultaneous optimization of the background parameters. The background was simulated using a combination of a Shirley and a Tougaard background. The binding energies (BE) were corrected for the charge shift using the C 1s peak of graphitic carbon (BE = 284.8 eV) as a reference.

Gas sensor tests were performed by in situ conductivity measurements in an automatic set up with a flow chamber. The sensor resistance was measured at 1.3 V DC-voltage in situ under a controlled gas flow of 100 ± 0.1 mL/min at a temperature fixed in the range of 400–550 °C. Purified air with a pre-assigned humidity (RH = 0% and RH = 30% at 25 °C) was used as a background gas. The test gases containing CO (20 ppm) and NH_3_ (20 ppm) were created from certified gas mixtures by the dilution with purified air with a pre-assigned humidity The corresponding gas flows were controlled by electronic mass-flow controllers (Bronkhorst).

## Supporting Information

Survey X-ray photoelectron spectra of SiC, ZnO, ZnO/SiC_15 nanocomposite, and X-ray photoelectron spectra of ZnO/SiC_15 nanocomposite in the Si 2p region.

File 1 XPS data.
