# Supplementary material for: High-temperature resistive gas sensors based on ZnO/SiC nanocomposites
Source: Beilstein J Nanotechnol. 2019 Jul 26;10:1537–47. doi: 10.3762/bjnano.10.151 (PMC6664407; doi:10.3762/bjnano.10.151)
Supplement: File 1 — XPS data. [file Beilstein_J_Nanotechnol-10-1537-s001.pdf]

BEILSTEIN JOURNAL OF NANOTECHNOLOGY

Supporting Information

for

High-temperature resistive gas sensors based on ZnO/SiC nanocomposites

Vadim B. Platonov, Marina N. Rumyantseva, Alexander S. Frolov, Alexey D. Yapryntsev and Alexander M. Gaskov

Beilstein J. Nanotechnol. **2019,** *10,* 1537–1547. doi:10.3762/bjnano.10.151

XPS data

License and Terms: This is a supporting information file under the terms of the Creative Commons Attribution License (http://creativecommons.org/licenses/by/4.0). Please note that the reuse, redistribution and reproduction in particular requires that the authors and source are credited.
The license is subject to the *Beilstein Journal of Nanotechnology* terms and conditions: (https://www.beilstein-journals.org/bjnano)

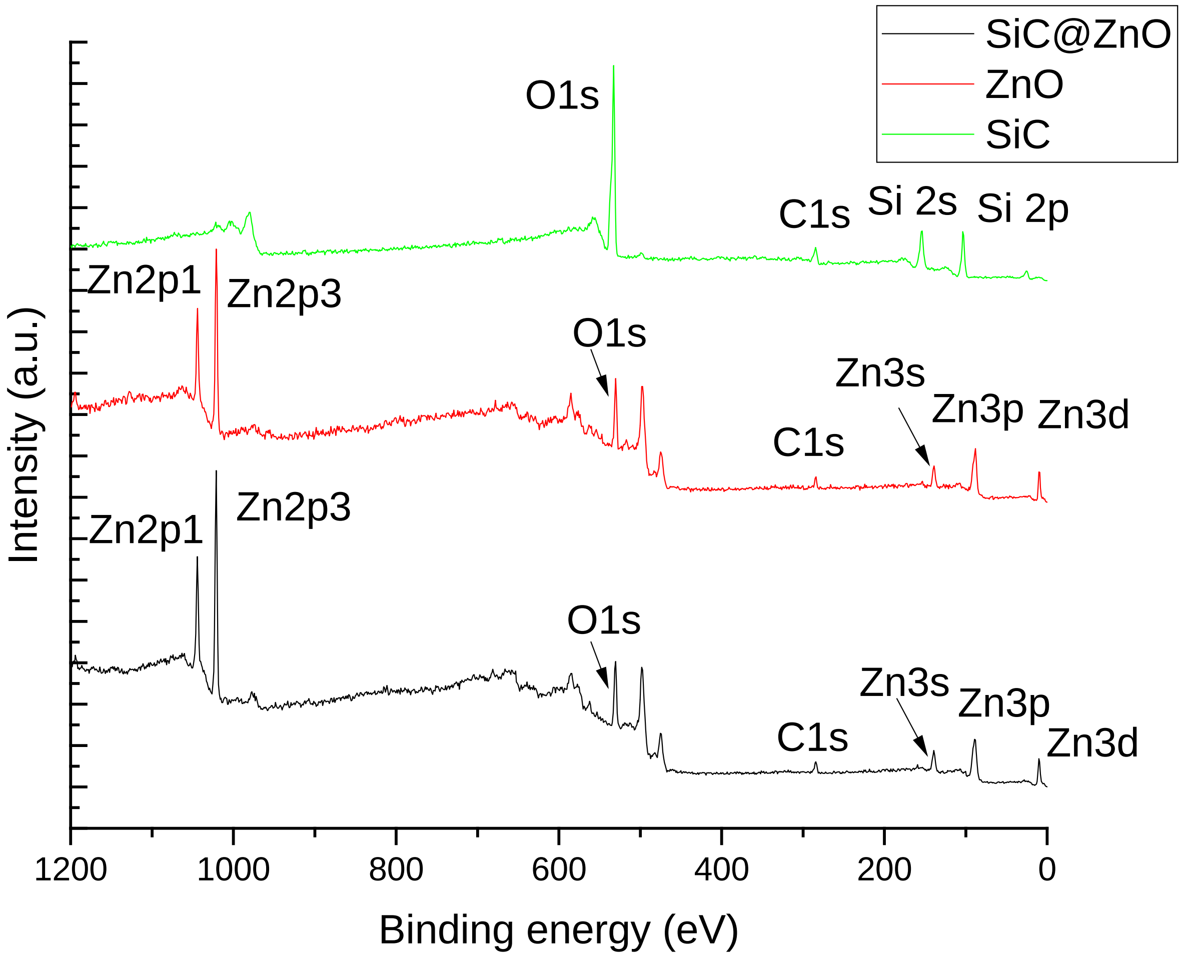

Figure S1: Survey X-ray photoelectron spectra of SiC, ZnO, ZnO/SiC_15 nanocomposite.

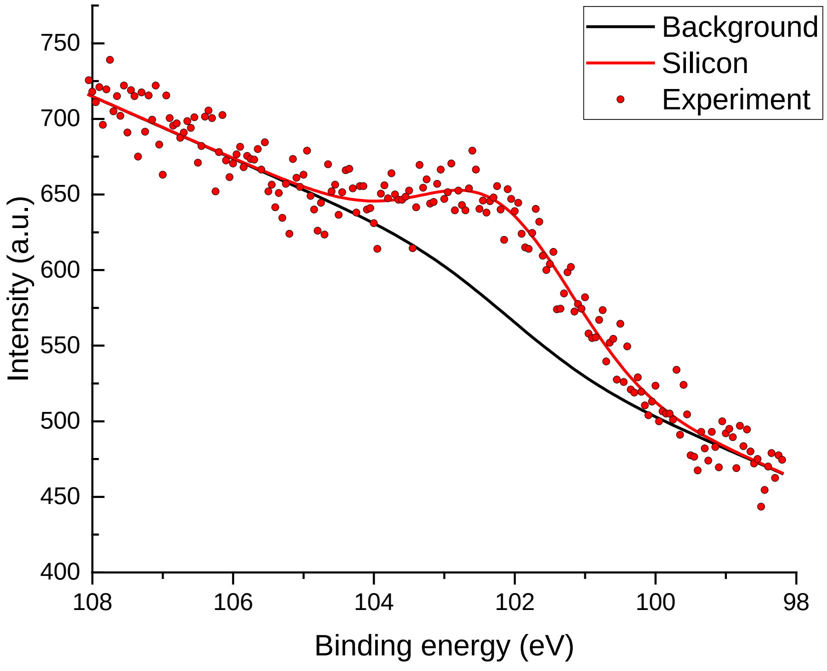

Figure S2: X-ray photoelectron spectra of ZnO/SiC_15 nanocomposite in the Si 2p region.
